# Development and Recent Progress of Hoses for Cryogenic Liquid Transportation

**DOI:** 10.3390/polym16070905

**Published:** 2024-03-26

**Authors:** Qiang Chen, Qingguo Sun, Jia Yan, Yunguang Cui, Lufeng Yang, Xiaojing Yang, Zhanjun Wu

**Affiliations:** 1 State Key Laboratory of Technology in Space Cryogenic Propellants, Beijing Special Engineering Design and Research Institute, Beijing 100028, China; chan1629@163.com (Q.C.); 13601380071@163.com (Q.S.); ylf0801@126.com (L.Y.); suruo1112@sina.com (X.Y.); 2 School of Materials Science and Engineering, Dalian University of Technology, Dalian 116024, China; 3 School of Mechanics and Aerospace Engineering, Dalian University of Technology, Dalian 116024, China; cyg1363956827@163.com

**Keywords:** cryogenic hoses, liquefied natural gas, liquid oxygen, cryogenic liquid transportation

## Abstract

Recently, the application of cryogenic hoses in the field of cryogenic media has become a hot topic, especially in the industry of offshore liquefied natural gas and aerospace field. However, the structure of cryogenic hoses is complex, and reasonable structural properties are required due to the harsh working conditions. There is still plenty of scope for further development to improve the performance in all aspects. In this paper, the current development status of cryogenic hoses for liquefied natural gas (LNG) transportation is reviewed first, including the types, manufacturers, structural forms, performance, and key technical challenges. And then, the recent progress and prospect of cryogenic hoses for cryogenic liquid transportation (such as LNG and liquid oxygen) are summarized, including structure design, low-temperature resistant polymers, liquid oxygen compatible polymers, and leakage monitoring technologies. This paper provides a comprehensive overview of the research development and application of cryogenic hoses. Moreover, future research directions have been proposed to facilitate its practical applications in aerospace.

## 1. Introduction

Cryogenic hoses are extensively utilized in the aerospace, maritime transportation, and other industries for the transportation and refueling systems of cryogenic liquid fuels or media. These hoses are mainly divided into two major types, including metal corrugated hoses and cryogenic composite hoses based on thermoplastic materials and metal reinforcement wires [1,2]. Metal corrugated hoses possess the advantages of mature technology, low costs, and high safety, so it is widely used [3,4]. However, they have drawbacks such as heavy weight, large bending radius, low flexibility, and difficulty in aligning during pipe connections [5,6]. On the other hand, cryogenic composite hoses, with their lightweight nature, small bending radius, and easy pipe connections, effectively address the challenges associated with metal hoses [7,8]. However, the reliability issues due to the low-temperature embrittlement of polymer materials and compatibility problems with certain super low-temperature media such as liquid oxygen (LOX) restrict their applications in the field of super low-temperature media like liquid hydrogen and liquid oxygen.

Currently, cryogenic composite hoses are only gradually used in offshore liquefied natural gas (LNG, −162 °C) systems [9]. In the field of offshore LNG, Metal corrugated hoses are only competent for offshore LNG operations in favorable sea conditions, greatly restricting operational times [10,11]. In contrast, cryogenic composite hoses, known for their flexibility, corrosion resistance, lightweight, and easy operation, have been increasingly employed in offshore LNG systems [12,13,14]. This allows for the safe completion of LNG transport and loading/unloading operations between ships and platforms even in harsh sea conditions [15,16,17].

At present, many businesses and scholars have launched the research and development of cryogenic hoses. There are many models and types of cryogenic hoses and each has its own characteristic. In this paper, we provide a detailed introduction to the products of different businesses, including the types, structural forms, structural characteristics, and key technical challenges. At the same time, we pointed out some technical challenges and also proposed corresponding solutions. In addition, we further explored the possibility of the development of cryogenic composite hoses for liquid oxygen (LOX). And, the testing of some materials that might be used in cryogenic composite hoses for LOX has been completed, which lays the foundation for further research. Finally, some suggestions are given about the particular directions for further research and advancement.

## 2. The Types of Cryogenic Hoses

Cryogenic hoses can be categorized according to the transported media, including LNG hoses, liquid oxygen hoses, liquid hydrogen hoses, liquid nitrogen hoses, and so on. Currently, Cryogenic hoses designed for the high-flow transport of cryogenic liquids are primarily applied to offshore LNG transport. The design and manufacturing of cryogenic hoses for LNG operations at sea are mainly guided by the BS EN ISO 20257 [18,19]. This standard outlines the design requirements and test criteria that LNG hoses must fulfill. The cryogenic hoses used for LNG are divided into two main types based on the structural form: one is metal corrugated hoses and another with the wall made of polymer films and polymer braided materials wound into a multilayer wall structure called cryogenic composite hoses [7]. The metal corrugated hoses are further categorized into reinforced metal corrugated hoses and high vacuum metal corrugated hoses, specifically [20].

### 2.1. The Reinforced Metal Corrugated Hoses

**Figure 1:**
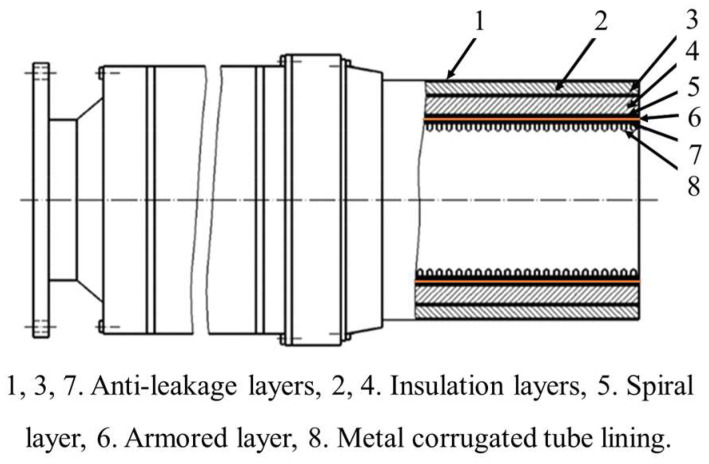
Typical structure of reinforced metal corrugated hose [24].

According to the specifications of EN1474-2-2008, reinforced metal corrugated hoses are primarily composed of three anti-leakage layers, two insulation layers, an armored layer, a spiral layer, and a metal corrugated tube lining layer [13], as shown in Figure 1. The configuration, the number of layers, and the stacking method of these layers can be determined based on the specific working conditions. The lining layer, a thin-walled metal corrugated tube made of stainless steel solves leakage issues and provides support for the internal pressure loads. The armored layer not only bears axial loads but also provides a certain level of insulation [21,22]. The spiral layer serves to determine the position of the armored layer and offers radial support. To effectively prevent heat transfer, at least one insulation layer is needed to act as thermal protection [23]. These insulation layers are helpful to prevent the external icing of the hoses during work and ensure that the internal temperature of the hose is stabilized.

**Figure 2:**
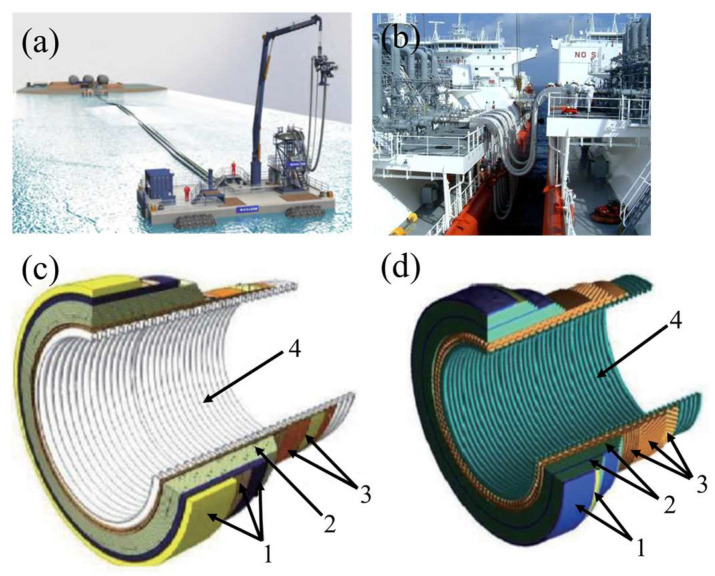
Two types of reinforced metal corrugated hoses by Technip [25]: (**a**) Floating type and (**c**) its structure; (**b**) Suspended type and (**d**) its structure (The performance of layers is listed in Table 1).

In 2008, the French company Technip developed two forms of LNG-reinforced metal corrugated hoses, namely the floating type and the suspended type corrugated hoses as shown in Figure 2a,b, respectively, according to the actual conditions of offshore operations. The main structure includes a metal lining tube, an armored layer, an insulation layer, and an outer protective layer [25,26,27,28]. The specific structure is shown in Figure 2c,d, and the layers along with their specific functions are listed in Table 1 [29]. The cryogenic hoses designed and produced by Technip have an inner diameter of 16 inches and an outer diameter of 17 inches, their maximum working pressure is 1.8 MPa, the medium transmission design velocity is 7 m/s, and the designed service life is up to 10 years. These two kinds of hoses were certified by Bureau Veritas (BV) in 2010 and 2015, respectively [30]. The overall flexibility of Technip’s LNG hoses is slightly lower, and the minimum achievable bending radius is relatively large. However, the use of corrugated inner tubes ensures good sealing and high strength. In addition, the insulation layer provides effective thermal insulation and prevents icing on the outer wall of the hose.

### 2.2. The High Vacuum Metal Corrugated Hoses

**Figure 3:**
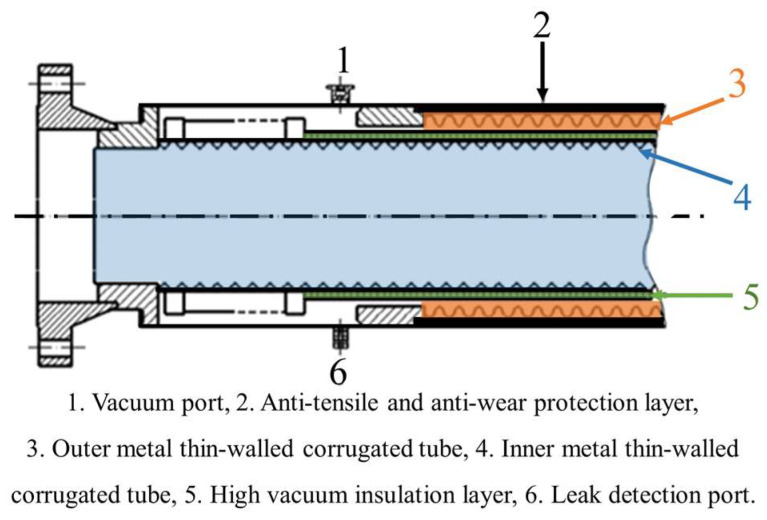
Typical structure of high vacuum type metal corrugated hoses [24,32].

The high vacuum type metal corrugated hoses adopt a “pipe in pipe” structural form, mainly consisting of inner and outer metal thin-walled corrugated tube, anti-tensile and anti-wear protection layer, high vacuum insulation layer, vacuum port, and leak detection port [31,32]. The typical structure of a high vacuum-type metal corrugated hose is shown in Figure 3. The basic principle involves supporting the inner and outer metal thin-walled corrugated tubes with spacers, creating a certain annular gap. This annular gap is then evacuated to form a super-insulated vacuum layer, ensuring the hose’s insulation performance. Additionally, corresponding sensors are installed in the annular gap to determine whether there is leakage by monitoring pressure or temperature changes within the gap.

**Figure 4:**
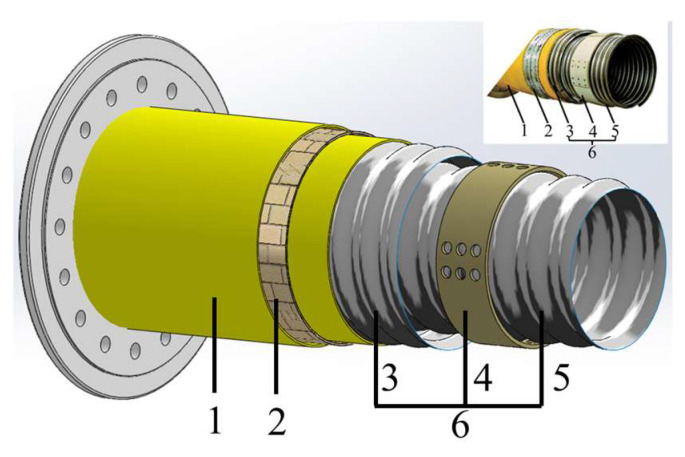
The structure of Nexans high vacuum metal corrugated hose [32] (The performance of layers is listed in Table 2).

Nexans company in France started to develop LNG high vacuum metal corrugated hoses in 2000, and the final product is suitable for use in relatively good sea conditions [33]. Its structure is shown in Figure 4, mainly consisting of internal and external metal thin-walled corrugated tubes, annular spacers between metal corrugated tubes, an insulation layer, an armored layer, and an outer protective layer. The structure and corresponding performance of each layer are listed in Table 2.

This high vacuum metal corrugated hose utilizes a spiral metal corrugated tube, which is simple to manufacture, offers excellent sealing performance, and has high strength. During normal operation, it provides effective insulation, preventing the occurrence of ice on the outer wall of the hose. However, it lacks overall flexibility, and the achievable minimum bending radius is limited. Therefore, it is suitable for use in relatively good sea conditions. However, the using of high vacuum insulation comes with higher costs, and in the event of a leak, the insulation effect can be instantly compromised.

### 2.3. The Cryogenic Composite Hoses

#### 2.3.1. The Cryogenic Composite Hoses for Liquefied Natural Gas (LNG)

Cryogenic composite hoses are wound by multiple layers of polymer films and polymer fiber braids tightened by inner and outer helical metal wires to create a sealed tubular structure [12,23]. The polymer film layers prevent leakage during medium transport, the polymer braiding layers enhance the axial and radial strength, and the inner and outer helical metal wires can provide skeletal support while enhancing the strength of the cryogenic composite hoses [12,24]. The inner diameter, screw pitch, film type and thickness, number of winding layers, and the specification of inner and outer reinforcing wires of the cryogenic composite hoses should be designed according to the requirements of the actual working conditions. Depending on the liquid medium being transported, the choice of polymer films varies. The cryogenic mechanical properties, the compatibility of these films with the medium, and the barrier properties against liquid media need to be considered. At present, cryogenic composite hoses are mostly used for the transmission of LNG at sea.

**Figure 5:**
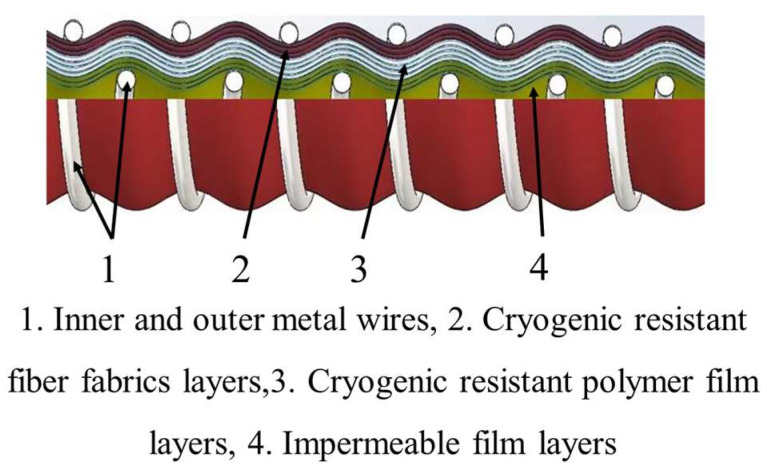
Schematic of the structure of cryogenic composite hoses by Japan Meiji company.

The 0933 series cryogenic composite hoses manufactured by Japan Meiji Hose company (MEIJIFLEX Hose) [35] located in Osaka, Japan, with a working temperature range of −200 °C to +80 °C, are designed for the transport of liquid nitrogen (−196 °C) and LNG (−162 °C), as shown in Figure 5. Its internal structure consists of multiple cryogenic-resistant polymer films and fiber fabrics, layered and wound to meet the cryogenic mechanical performance requirements of the operating environment. The film layers primarily serve the purpose of sealing and preventing leakage, while the fiber fabrics are employed to enhance the structural strength of the cryogenic composite hoses. The manufacturing process of this kind of hose is simple and automated, leading to a low cost. Meanwhile, a heat-reflective layer is coated on the surface of film layers to enhance the thermal insulation to a certain extent. However, the thermal insulation performance with this method is limiting and needs to be further improved. The cryogenic composite hoses made by Japan Meiji are mainly used for the transportation of marine LNG, exhibiting strong adaptability to various harsh marine environments, which can significantly improve transporting efficiency.

**Figure 6:**
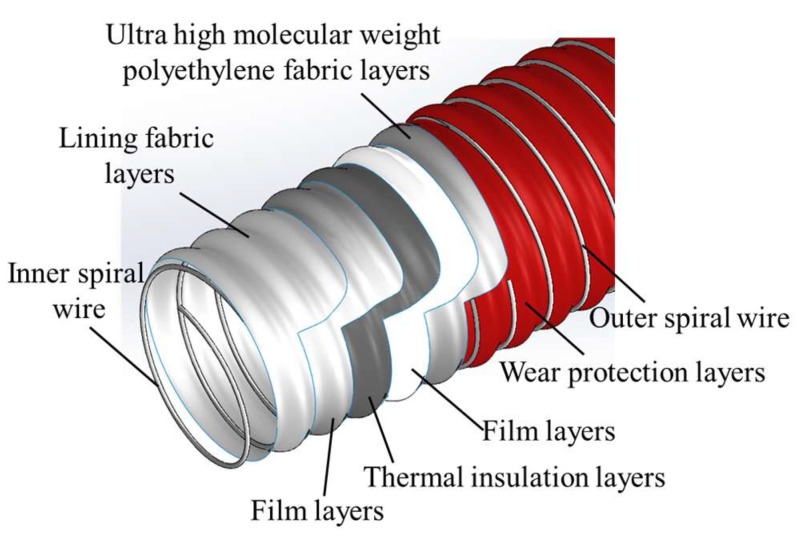
The structure of composite hoses by Dunlop.

The Dunlop company in the UK has been dedicated to the research and development of cryogenic composite hoses used for LNG since 2011, which are lightweight, high-strength, and flexible [36]. They introduced a new cryogenic composite hose used for LNG as shown in Figure 6, which underwent various performance tests at normal and cryogenic temperatures, ultimately receiving DNV certification in 2016 [9]. The design form of this cryogenic composite hose offers excellent flexibility, a smaller bending radius, and can adapt to relatively harsh sea conditions. By adding the thermal insulation layers, it effectively prevents the outer surface of the hose from icing in a short period. However, the insulation layers are located inside of the pipe wall, and it poses manufacturing challenges, increasing the difficulty of processing and requiring strict control over the pre-compression force between the winding layers.

**Figure 7:**
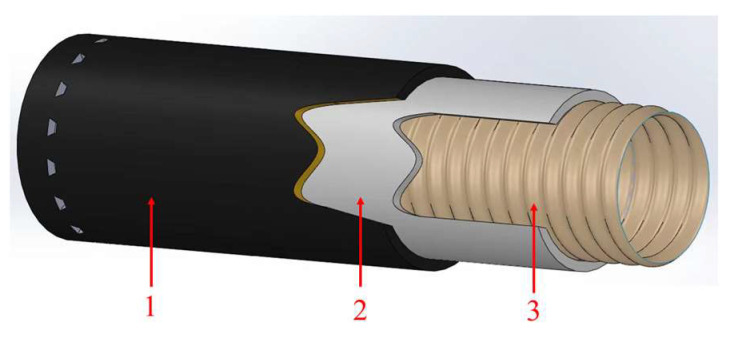
Structure of cryo-line composite hose [38,39] (The performance of layers is listed in Table 3).

During the transportation of cryogenic fluids, there is a high demand for the thermal insulation performance of cryogenic composite hoses to prevent the sudden increase of inner pressure due to liquid vaporization. In conclusion, cryogenic composite hoses need additional insulation layers to prevent frosting outside of the hose and meet the insulation requirement. Sweden’s Trelleborg and France’s Total developed a cryogenic composite hose named Cryo-line in 2016, based on the “pipe in pipe” concept as shown in Figure 7. After static and dynamic pressure testing at normal and low temperatures, the cryogenic composite hoses successfully obtained BV certification [37,38,39]. The composition and performance characteristics of each layer of this hose are listed in Table 3. The cryogenic composite hoses have improved insulation performance, effectively preventing the outer surface of the hose from icing. Additionally, a leak detection system, a monitoring method based on fiber optic detection technology, is embedded in the insulation layers to improve the safety of the cryogenic hoses during work. However, the presence of the insulation layers and monitoring system makes the manufacturing process complex and increases the cost.

#### 2.3.2. The Cryogenic Composite Hoses for Liquid Oxygen

Compared to the cryogenic composite hoses used for LNG, those used for liquid oxygen transportation face a more severe and extreme working environment, posing greater challenges in design and manufacturing [41]. For the safe use of liquid oxygen hoses, two crucial challenges must be addressed and overcome. Firstly, the temperature of liquid oxygen (−183 °C) is lower, leading to more severe low-temperature embrittlement of polymer materials and heightened safety concerns [42]. Secondly, Due to the strong oxidizing property of liquid oxygen (LOX), most polymers possessing excellent cryogenic mechanical properties are LOX incompatible [43,44]. And, if these incompatible polymers are used as the inner layer material of the hose, it probably leads to significant safety accidents such as combustion and even explosion under certain external energy stimulation [44,45,46]. Therefore, the issue of material compatibility with liquid oxygen is a crucial problem that needs to be solved urgently.

**Figure 8:**
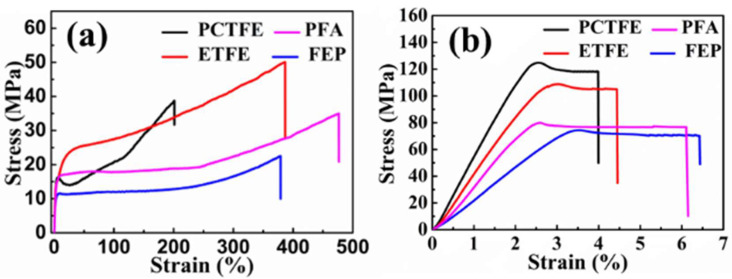
Tensile stress–strain curves of PCTFE, PFA, ETFE, and FEP plastic films under (**a**) room temperature and (**b**) liquid nitrogen temperature (−196 °C).

It is found that most metals (except titanium alloys) exhibit good compatibility with liquid oxygen, including stainless steel, aluminum and its alloys, copper and its alloys, and nickel and its alloys [47]. Therefore, vacuum metal corrugated hoses are widely used for liquid oxygen transportation currently. However, few polymers are compatible with liquid oxygen. At present, among thermoplastic polymer materials, only some types of fluoropolymers are LOX compatible [48]. Research shows that some fluoropolymers, such as polytetrafluoroethylene (PTFE), polychlorotrifluoroethylene (PCTFE), and ethylene tetrafluoroethylene copolymer (ETFE), are commonly used as sealing materials for liquid oxygen pipe fittings [49,50,51,52], which indicated that these fluoropolymers are compatible with LOX. In this section, we investigated the mechanical properties of several fluoroplastic films available on the market, including perfluoroalkoxy polymers (PFA), PTFE, PCTFE, and ETFE. And conducted liquid oxygen impact sensitivity tests on these four fluoroplastic films according to the method specified in the ASTM D2512-95 standard [53]. The results show that they are all compatible with liquid oxygen. At the same time, we also tested their mechanical properties at room temperature and liquid nitrogen temperature (−196 °C) (following the standard ISO-527-3) [54], and the tensile stress–strain curves of these four plastic films are shown in Figure 8. It can be seen that although the tensile strength of plastic films increases at low temperatures, the elongation at break decreases exponentially, ultimately resulting in a sharp decrease in fracture strain energy, indicating that the material becomes less ductile and more brittle at ultra-low temperatures, compared to room temperature.

The values of tensile strength, elongation at break, and elastic modulus for the four fluoropolymer films at room temperature and liquid nitrogen temperature (−196 °C) were listed in Table 4. At the temperature of liquid nitrogen (−196 °C), the tensile strength of PCTFE, ETFE, FEP, and PFA films is approximately 7.8, 4, 6.2, and 4.8 times higher than that at room temperature, respectively, and the elastic modulus is approximately 4.2, 2.9, 4.4, and 5.7 times higher, indicating a significant increase in material strength and stiffness at the cryogenic environment. Meanwhile, the elongation at break of the four types of films at −196 °C shows a reduction of orders of magnitude compared to that at room temperature. Therefore, although these four types of fluoropolymer films are liquid oxygen compatible and exhibit a certain level of strength at ultra-low temperatures, it is challenging for them to meet the pressure-resistant requirement in hoses for liquid oxygen transport. In order to enhance the safety and flexibility of liquid oxygen hoses, these fluoropolymer films need to be combined with other low-temperature-resistant polymeric films such as polyimide, ultra-high molecular weight polyethylene, or polyester.

#### 2.3.3. The Manufacturing Process of Cryogenic Composite Hose

**Figure 9:**
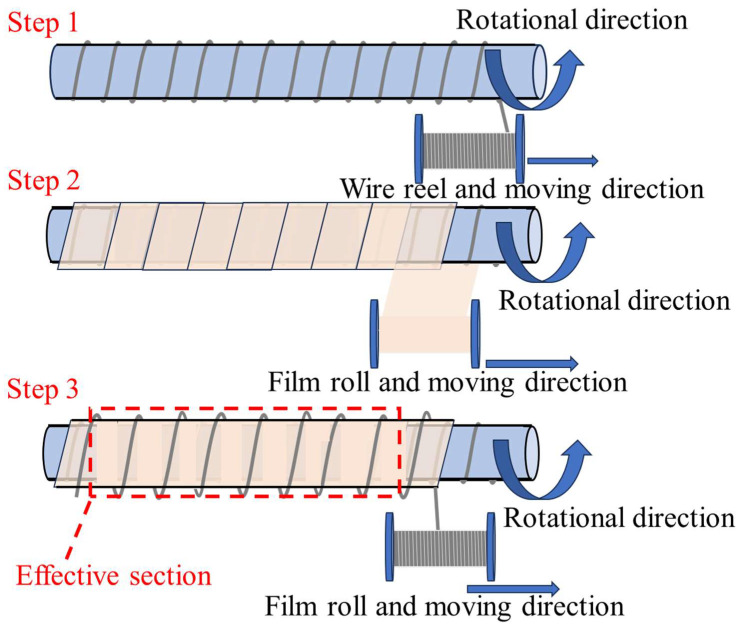
The schematic diagram of the manufacturing process.

The properties of the medium conveyed by the cryogenic composite hoses determine the use of materials. After the material is determined, the cryogenic composite hoses can be processed and manufactured according to the designed structural form and structural parameters. The entire manufacturing process of the wall of cryogenic composite hose is completed on a rotating mechanical shaft, as shown in Figure 9. The diameter of the mechanical shaft determines the inner diameter of the cryogenic composite hoses. The specific manufacturing process is summarized as follows:

First, the inner wire is wound onto the mechanical shaft according to the screw pitch size of the cryogenic composite hoses. Then, each film, fabric, and other thermal insulation material is wrapped around the wire according to the designed angle in the lay-up sequence. Finally, all layers are laid, the outer wire is wrapped around the outside at the same pitch, and half a screw pitch apart from the inner wire to form a self-locking structure. After the completion of the body of the cryogenic composite hose is removed from the rotary shaft, intercepting the valid section and installing the connectors. The above is the simplified manufacturing process of cryogenic composite hose, only for the reader to understand.

## 3. The Key Technical Challenges of Cryogenic Composite Hoses

### 3.1. The Material Selection for Cryogenic Composite Hoses

The selection of the material for cryogenic hoses is one of the crucial factors determining hose safety, fatigue resistance, and lifespan. For metal corrugated hoses, 316 L stainless steel exhibits good low-temperature toughness and corrosion resistance [31,55], it is suitable as the material used in metal corrugated hoses for the transportation of most low-temperature media. As for cryogenic composite hoses, when the low-temperature fluid medium exhibits strong oxidizing or corrosive properties, the material selection of the conveying hose is critical. Especially in the selection of the inner layer material in direct contact with the medium, the material must not only consider the mechanical properties (strength, flexibility, mechanical fatigue performance, etc.) [44,56,57] but also consider the barrier, oxidation resistance, and corrosion resistance under the corresponding medium.

Due to the small molecular weight of some media (such as liquid hydrogen), for a period of time, it will permeate out of the container wall, making the hydrogen content of the air increase, which is prone to accidents [58,59,60]. Therefore, it is necessary to perform a penetration test on the materials used for this medium. In addition, there are some media with strong oxidation and corrosion (such as liquid oxygen), and some polymer materials will oxidize in liquid oxygen for a long time, thereby reducing their own mechanical properties. Therefore, the corresponding material needs to be soaked in liquid oxygen test, and then the mechanical properties of the soaked material are tested. Therefore, there are two methods for material selection: First, material performance tests need to be completed according to the actual situation based on the existing materials, and then applied to the cryogenic composite hoses after meeting the requirements. Second, combined with the performance characteristics of existing materials, the synthesis of high-performance new materials that meet the requirements, and then through various performance tests to ensure that they can meet the requirements of cryogenic composite hoses.

### 3.2. The Structure Design and Numerical Simulation of Cryogenic Composite Hose

Due to the harsh operating conditions of cryogenic hoses, the structural design must consider the impact of temperature, internal pressure, bending, and torsion on its mechanical properties [1,13,55]. Additionally, factors such as thermal insulation performance, sealing performance, flexibility, strength, and weight must be taken into account [14]. The design of the hose aims to ensure safety and reliability while obtaining reasonable structural forms, interlayer thicknesses, and stacking methods for each layer. Furthermore, the structural analysis of cryogenic composite hoses involves winding structure analysis, modeling analysis of multilayer non-bonded composite structures, and modeling analysis of braided structures [61]. These include numerical simulations of structural and thermal-solid coupling, addressing challenges in unit analysis, interlayer contact analysis, nonlinear problem analysis, and constraints on boundary conditions [31,62]. In summary, the structural design and modeling analysis of cryogenic hoses need to consider the mechanical response characteristics under different conditions and balance various performance parameters for optimal structural design. The validation process involves structural numerical simulations, nonlinear analysis of interlayer structures, fluid–solid coupling calculations, and heat transfer calculations to obtain a low-temperature hose structure suitable for specific operating conditions [7]. The future research aim is to overcome the above problems to obtain lightweight, high-strength, high-toughness, and large-size cryogenic composite hoses that meet various working conditions.

### 3.3. The Insulation Measures of Cryogenic Composite Hoses

There are three heat transfer styles, conduction, convection, and radiation. It is a challenge to achieve absolute insulation within limited thickness within the millimeter or centimeter scale range [63,64]. For cryogenic hoses, two methods are usually used to achieve thermal insulation, by vacuum interlayer between inner and outer walls or by insulation materials. The former provides excellent insulation performance but is associated with poor flexibility and higher overall costs. Additionally, Once the vacuum layer is damaged, the insulation performance will be immediately lost resulting in the failure of the entire hose structure. The insulation layers of composite hoses need to meet high requirements, such as lightweight, excellent flexibility, and longer service life. There are many kinds of traditional insulation materials, including glass wool, foam asbestos, aluminum silicate fiber felt, and polyurethane. They are usually used in the form of pads or plates for insulation protection outside vessels or buildings [65,66,67]. Flexible aerogel is a new type of insulation material, the biggest advantage is that it has flexibility, and can be used for insulation measures of different curvature surfaces [68,69].

The insulation performance of common insulation materials is listed in Table 5. At present, aerogel possesses a promising prospect in cryogenic composite hoses, due to its lightweight, excellent, and stable insulation performance, low-temperature resistance, and long insulation life [70]. As early as the late 1980s, researchers in the Lawrence Livermore National Laboratory (LLNL) prepared the lightest silica aerogel with a density of 0.003 g/cm^3^ and only three times than air. At room temperature and pressure, its thermal conductivity ranges between 0.011 W/(m·K) and 0.016 W/(m·K) [71]. If aerogel is used as insulation material for cryogenic composite hoses, it can effectively reduce the wall thickness and overall weight. The reinforced metal corrugated hose developed by Technip in France uses aerogel as the insulation material, achieving excellent insulation performance and effectively preventing the outer wall from icing during the working process [16]. Thermal insulation performance is one of the important indexes of cryogenic composite hoses. Previous studies have shown that aerogel is an excellent insulation material, However, further research is needed to realize its application in cryogenic composite hoses, especially how to reasonably apply it to the structure of cryogenic composite hose and ensure its fault tolerance.

### 3.4. The Leak Monitoring System of Cryogenic Composite Hoses

**Figure 10:**
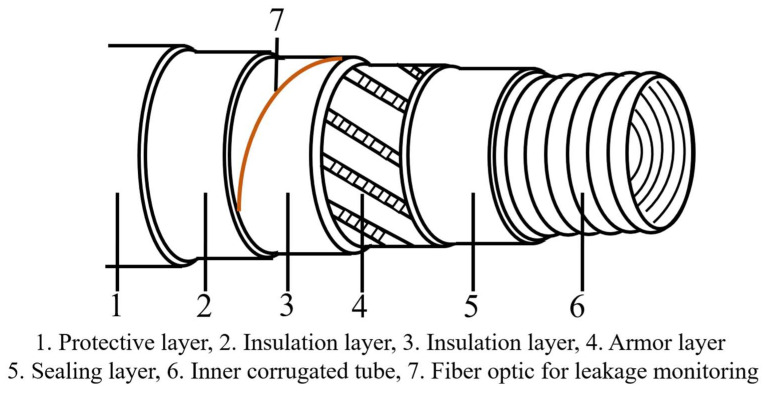
The cryogenic composite hoses with an optical fiber monitoring system [25].

In the working process, the real-time monitoring of the positions prone to leakage and fatigue in cryogenic hoses can provide more effective safety assurance throughout the entire operation. Fiber optic sensing systems offer advantages such as resistance to electromagnetic interference, high insulation, and reliable sensitivity, they can provide real-time monitoring during the working of cryogenic hoses so that leaks can be detected timely to prevent accidents [25,77,78]. The leak detection system for cryogenic hoses based on fiber optics is illustrated in Figure 10. The challenges of the application of fiber optic monitoring systems in cryogenic hoses are mainly the distribution of fiber optic systems, service life, and bending flexibility with the complex working conditions of cryogenic hoses. Reasonable optical fiber arrangement can realize all-round monitoring, improve the fault tolerance rate of optical fiber monitoring systems can greatly improve the service life of cryogenic composite hoses, and indirectly reduce the production cost.

## 4. Summary and Perspectives

Compared to metal corrugated hoses, flexible composite hoses based on thermoplastic polymers have the advantages of being lightweight, good flexibility, simple operation, and easy connection. With the continuous development of the offshore LNG bunkering market, the superiority of flexible composite hose bunkering methods has become increasingly prominent. These hoses can be operated in bad and extreme sea conditions, breaking the limitation of traditional ones which can only be used in good sea conditions, significantly reducing the operational risk and improving working efficiency. Additionally, with the continuous development in various industries, there is a substantial potential demand for the transport and bunkering systems of low-temperature media such as liquid oxygen, liquid hydrogen, and LNG. Therefore, there is a wide prospect for cryogenic composite hoses applied in LNG transport and ship bunkering systems, liquid oxygen or liquid hydrogen bunkering systems for rockets, as well as commercial cryogenic media transfer and bunkering systems.

Due to the extreme working conditions, such as ultra-low temperature and high pressure, there are high requirements for the weight, cryogenic mechanical properties, chemical stability, leak resistance, thermal fatigue resistance, and thermal insulation performance of cryogenic composite hoses. It makes material selection, structural design, manufacture, safety monitoring, and testing of cryogenic composite hoses face many challenges. First of all, materials are fundamental to the manufacture of cryogenic composite hoses. cryogenic and corrosion-resistant polymer materials are the key to limiting the further development of cryogenic composite hoses. In addition, the reasonable structural design can improve the service life and working strength of the low-temperature composite hose, it also reduces the weight and improves the toughness as much as possible under the premise of ensuring sufficient strength. Finally, reliable monitoring technology can avoid risks in advance to ensure the safe operation of the cryogenic composite hose. The further development of cryogenic composite hoses first requires efforts in the research and development of high-performance materials. Then, combined with accurate numerical simulation, the interface mechanism between different layers should be considered comprehensively, and reasonable winding Angle, layering sequence, pitch, wave height, and wave width need to be optimized, and finally the structure form with excellent performance is obtained. Finally, in order to ensure the safety and reliability of cryogenic composite hoses, the application of detection technology also needs further research. Overcoming the above challenges will break the shackles of cryogenic composite hoses and further improve the efficiency of offshore natural gas development and aerospace cryogenic propellant filling.

In the future, the technical developments in structure/function integrated materials, optimization methods and software for hose structures, intelligent manufacturing technology, novel sealing connection structures, and health monitoring technology will lead the continuous development of cryogenic composite hoses towards directions of lightweight, high flexibility and safety, long life, large size, and self-monitor.

## Figures and Tables

**Table 1:** Structure and Performance of reinforced metal corrugated hoses by Technip [25,29].

Number	Layer Type	Performance Characteristics
1	Outer protective layer	1. It prevents corrosion and wear of critical materials on the hoses wall from external environments. 2. The outer protective layers of the floating type hoses are wound by thermoplastic elastic material, while the suspended type hoses are wrapped by self-adhesive tape.
2	Insulation layer	1. It prevents heat transfer, avoiding icing on the outer wall of the hoses and gasifying of the medium inside the hoses. 2. The insulation layers of the floating type hoses are wrapped by aerogel foam tape, while the suspended type hoses are wrapped by polyethylene foam tape.
3	Armored layer	1. It is placed between the insulation layers and the metal inner tube, primarily bears axial loads, enhances the hose’s axial tensile strength. 2. It is wrapped by a double-layer of polyester fiber fabric. 3. The armored layer of the floating type hose is additionally equipped with wear-resistant strips and flat steel strips. 4. The armored layer of the the suspended type is wrapped with nylon braided tape.
4	Metal inner tube	1. It is a thin-walled corrugated tube made of 316 L stainless steel. 2. It provids skeletal support and determins the inner diameter of hoses. 3. It is capable to withstand internal pressure loads during working.

**Table 2:** The structure and performance of Nexans high vacuum metal corrugated hose [32,34].

Number	Layer Type	Performance Characteristics
1	Outer protective layer	1. It prevents critical materials of hoses from wear or corrosion. 2. It is wrapped by thermoplastic polyethylene films according to the requirements.
2	Armored layer	1. It protects the metal thin-walled corrugated tube, 2. It provides sufficient axial rigidity and tensile strength for the hose. 3. It is wound by two layers of flat steel strips.
3	Outer metal corrugated tube	1. It is a part of the vacuum layer and bears some axial loads. 2. It is made of 316 L stainless steel.
4	Annular spacers	1. It supports the inner and outer metal thin-walled corrugated tubes to form the interlayer gap. 2. Its thickness within 0.1 mm and the thermal conductivity is low.
5	Inner metal corrugated tube	1. It is a part of the vacuum layer and bears some axial loads. 2. It provides skeletal support and determines the inner diameter of the hose. 3. It can withstand the internal pressure load during working. 4. It is made of 316 L stainless steel.
6	High vacuum insulation layer	1. It consists of components 3, 4, and 5. 2. It reduces heat convection and radiation. 3. There are 10 layers reflective foil are wound on the outer wall of the inner metal corrugated tube to ensur the excellent insulation performance.

**Table 3:** Structure and performance features of cryo-line composite hose [38,40].

Number	Layer Type	Performance Characteristics
1	Outer protective hose based on bonded flexible hose technology.	1. It prevents key materials of the hose from corrosion and wear. 2. The hose developed by Trelleborg company possesses good fatigue resistance.
2	Innovative and efficient insulation material + Leak monitoring system	1. The material is designed to reduce heat loss within the structure so that no ice will form on the outer cover of the cryogenic hoses. 2. The material have excellent properties over the full range of temperatures (from room to cryogenic temperature). 3. The leak monitoring system based on optical fiber technology is included in the annular space to check the evolution of temperature within the structure and prevent any abnormal activity during working.
3	Inner hose derived from composite hose technology	1. To achieve better sealing, this inner hose incorporates multiple polymeric film material and woven fabric material.

**Table 4:** The mechanical performance of four fluoropolymer at ambient and cryogenic temperatures.

	Performance	Tensile Strength/MPa		Elongation at Break/%		Elastic Modulus/GPa	
Material							
		20 °C	−196 °C	20 °C	−196 °C	20 °C	−196 °C
PCTFE		16.32 ± 1.02	127.78 ± 8.91	193.53 ± 9.01	4.01 ± 0.66	1.35 ± 0.10	5.65 ± 0.16
ETFE		25.04 ± 1.61	100.16 ± 5.03	386.71 ± 7.93	4.31 ± 0.26	1.69 ± 0.08	4.85 ± 0.21
FEP		11.52 ± 1.22	71.08 ± 4.87	379.04 ± 8.83	6.37 ± 0.19	0.59 ± 0.05	2.98 ± 0.20
PFA		16.85 ± 1.33	80.79 ± 8.43	478.89 ± 7.68	6.32 ± 0.29	0.58 ± 0.04	3.28 ± 0.27

**Table 5:** Performance comparison of common insulation materials [65,72,73,74,75,76].

Insulation Materials	Density (kg/m^3^)	Thermal Conductivity (W/m·K)	Operating Temperature (°C)	Advantages	Disadvantages
Glass wool	40~110	0.03~0.04	−120~800	Easy to form, low cost, and stable chemical properties	Prone to fiber debris
Foam asbestos	20~45	0.035~0.041	−50~400	Good low-temperature performance and sound absorption	High volume weight
Aluminum silicate fiber felt	70~120	0.034~0.12	<1000	Anti-seismic, Pressure-resistance and easy to form	Easy to produce dust
Polyurethane	30~70	0.035	<150	Long service life and damp-proof insulation	Brittle and splintery
Aerogel	100~170	0.011~0.016	−196~1000	Light, soft and Environmental	Easy to absorb water and high cost
